# Disease progression status during initial immune checkpoint inhibitor (ICI) affects the clinical outcome of ICI retreatment in advanced non‐small cell lung cancer patients

**DOI:** 10.1002/cam4.5939

**Published:** 2023-04-16

**Authors:** Masahiro Torasawa, Tatsuya Yoshida, Yuki Takeyasu, Yukiko Shimoda, Akiko Tateishi, Yuji Matsumoto, Ken Masuda, Yuki Shinno, Yusuke Okuma, Yasushi Goto, Hidehito Horinouchi, Noboru Yamamoto, Kazuhisa Takahashi, Yuichiro Ohe

**Affiliations:** ^1^ Department of Thoracic Oncology National Cancer Center Hospital Tokyo Japan; ^2^ Department of Respiratory Medicine Juntendo University Graduate School of Medicine Tokyo Japan; ^3^ Department of Experimental Therapeutics National Cancer Center Hospital Tokyo Japan

**Keywords:** immune checkpoint inhibitor, immune‐related adverse events, non‐small cell lung cancer, PD‐L1, retreatment

## Abstract

**Background:**

It is still unclear whether patients with advanced non‐small cell lung cancer (NSCLC), with disease progression after initial immune checkpoint inhibitor (ICI) therapy, would benefit from ICIs readministration.

**Patients and Methods:**

We retrospectively collected data from patients with advanced NSCLC who received ICI retreatment. Depending on the disease status at the discontinuation of the initial ICI therapy, the patients were divided into two groups: with disease progression (PD group) and without disease progression (Without PD group). Patients in the Without PD group were required to experience disease progression during the treatment‐free period. Efficacy was assessed by measuring the objective response rate (ORR) and progression‐free survival in retreatment (PFS‐R), while safety was assessed using the incidence of immune‐related adverse events (irAEs).

**Results:**

30 (46.7%) of 64 eligible patients were included in the PD group and 34 (53.1%) in the Without PD group. Patients in the Without PD group had better clinical outcomes than those in the PD group (ORR, 29.4% vs. 6.7%; *p* = 0.03, median PFS‐R, 4.1 months vs. 2.2 months, hazard ratio [HR], 0.61; 95% confidence interval [CI], 0.36–1.04; *p* = 0.07). Multivariate Cox regression analysis showed that patients in the Without PD group had significantly longer PFS‐R than those in the PD group (HR 0.42, 95% CI, 0.21–0.85; *p* = 0.015). In terms of safety, 28.1% of patients observed irAEs during ICI retreatment, and the incidence rate of grade 3 or higher irAEs was 7.8%. Specifically, of the 28 patients who discontinued their initial ICI treatment because of irAEs, 35.7% developed irAEs, and 28.6% experienced relapsed irAEs during ICI retreatment.

**Conclusion:**

Immune checkpoint inhibitor retreatment demonstrated efficacy in patients who discontinued initial ICI therapy for reasons other than disease progression. However, ICI retreatment was ineffective in patients with disease progression during the initial ICI treatment.

## 1 INTRODUCTION

Immune checkpoint inhibitors (ICI), which inhibit immune checkpoint proteins including programmed cell death 1 receptor (PD‐1), programmed death ligand 1 (PD‐L1), and cytotoxic T‐lymphocyte antigen‐4 (CTLA‐4), have improved the prognosis across various types of cancer. Several ICI (nivolumab, pembrolizumab, atezolizumab, and the combination of nivolumab plus ipilimumab) have been approved for the treatment of advanced non‐small cell lung cancer (NSCLC). In addition, durvalumab, an anti‐PD‐L1 antibody, has been approved as consolidation therapy after chemoradiation in locally advanced NSCLC.^1^ Although a subset of NSCLC patients can achieve long‐term clinical benefits when receiving ICI, most of them discontinue the therapy owing to disease progression or adverse events (AEs), especially immune‐related adverse events (irAEs).

Several studies^2^, ^3^, ^4^, ^5^, ^6^, ^7^, ^8^, ^9^, ^10^, ^11^, ^12^ have evaluated the efficacy and safety of ICI retreatment in patients with NSCLC. However, most of them have been retrospective and included a small number of patients. Currently, it is not well understood which population type of patients will benefit from the ICI retreatment. Furthermore, it remains unknown how the disease status at the time of initial ICI treatment discontinuation (whether discontinuation was a result of the disease progression or of events other than disease progression, such as irAEs development or clinical decision) affects the efficacy and safety of ICI retreatment. Santini et al. reported that clinical outcomes among patients with objective responses before a serious irAE were similar whether they were retreated or not.^12^ The National Comprehensive Cancer Network (NCCN) guidelines state that  ICI retreatment after a period of temporary discontinuation due to irAEs is not advisable in patients achieving confirmed response throughout the discontinuation period because the risk of AE recurrence can increase by the retreatment.^13^ Therefore, it is uncertain whether patients undergoing disease progression should be retreated with ICIs after the recovery of irAEs.

In this study, we aimed to evaluate the efficacy and safety of retreatment with ICIs in patients with NSCLC. We investigated how the disease status at the time of the initial ICI discontinuation affects the clinical outcomes of ICI retreatment.

## 2 PATIENTS AND METHODS

### 2.1 Patients and clinical characteristics

We retrospectively collected the clinical data of patients with advanced NSCLC who received at least one dose of ICI between April 2015 and August 2021 from a single center. The eligibility criteria for patients were as follows: (1) patients must have received at least two treatment lines of anti‐PD‐1/PD‐L1 antibody therapy during the course of the disease and (2) disease progression should be confirmed by computed tomography (CT) imaging before ICI readministration. The following patients were excluded: patients with primary resistance to initial ICI therapy, patients who were re‐treated with an ICI under investigation, and patients who received an ICI in combination with chemotherapy as retreatment.

**FIGURE 1:**
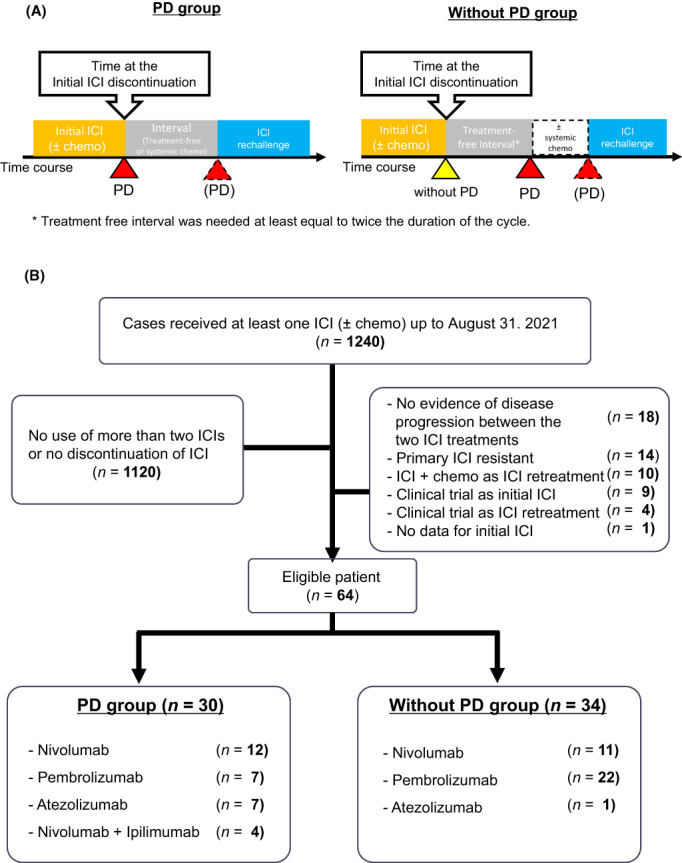
(A) Definition of ICI retreatment. (B) Patient flow. Chemo, chemotherapy; ICI, immune checkpoint inhibitor; PD, progressive disease.

Immune checkpoint inhibitor readministration was categorized into either “PD” or “Without PD” group (Figure 1A). “PD” group included patients who discontinued treatment owing to disease progression during the initial ICI treatment and subsequently received a second course of ICI therapy. “Without PD” group included patients whose initial ICI treatment was discontinued for any reason (irAEs or clinical decision) other than disease progression. Patients in the Without PD group were required to experience disease progression after a defined treatment‐free period (at least twice as long as the treatment cycle, e.g., at least 4 weeks for the twice‐weekly regimen).

Collected data included the following clinical characteristics: age, sex, Eastern Cooperative Oncology Group performance status (ECOG‐PS), smoking history, histology, epidermal growth factor receptor gene (*EGFR)* mutation status, clinical stage (according to the Union for International Cancer Control [UICC], 8th edition), PD‐L1 expression, history of chemotherapies, and radiation during ICI intervals. PD‐L1 expression was assessed by immunohistochemical staining (PD‐L1 immunohistochemistry [IHC] 22C3, pharmDx, Dako/Agilent) of the samples collected before the initial ICI treatment, and the PD‐L1 tumor proportion score (TPS) was calculated as the percentage of viable tumor cells with PD‐L1 positive staining. The study protocol was approved by the Ethics Committee of the National Cancer Center Hospital, Tokyo, Japan (approval no. 2015–355).

### 2.2 Outcome evaluation

The primary endpoints were to evaluate the objective response rate (ORR), progression‐free survival in retreatment (PFS‐R), and safety in the PD and Without PD groups. Antitumor efficacy was evaluated according to the Response Evaluation Criteria in Solid Tumors (RECIST) version 1.1.^14^ ORR was defined as the proportion of patients who achieved at least one complete (CR) or partial response (PR) after treatment. Patients without target lesions were included in one the following categories: CR, progressive disease (PD), or non‐CR/non‐PD. The disease control rate (DCR) was defined as the percentage of patients who achieved CR, PR, or stable disease (SD). In the case of SD, measurements must have met the SD criteria at least once at a minimum of 6 weeks after initiation of treatment. Treatment duration was between initial administration and the last treatment date. The median PFS for the initial ICI treatment was calculated from the first ICI dose to disease progression. Similarly, the median PFS‐R was defined as the time from the start of ICI retreatment to disease progression or death. Patients alive without a recorded date of progression at the last follow‐up were censored. Safety was assessed according to the US National Cancer Institute's criteria for the evaluation of adverse events (CTCAE), version 5.0,^15^ up to the time of disease progression. The data cut‐off date was November 30, 2021.

### 2.3 Statistical analysis

In the descriptive statistical analysis, the continuous variables were summarized by medians and ranges, while the categorical variables were summarized by frequency and percentage. We used Fisher's exact test to evaluate the differences in categorical data and either the t‐test or the Mann–Whitney *U* test for continuous data. PFS was analyzed using the Kaplan–Meier method,^16^ and differences were compared using the log‐rank test (two‐sided). The hazard ratios (HRs) and corresponding 95% confidence intervals (CIs) were estimated using the Cox proportional regression model.^17^ In the final model, *p*‐value <0.05 was considered significant. All collected data were analyzed using EZR statistical software, version 1.54^18^ (Saitama Medical Center, Jichi Medical University) and GraphPad Prism 9.0 (GraphPad). EZR is a graphical user interface (GUI) of R (R Foundation for Statistical Computing).

## 3 RESULTS

### 3.1 Patient characteristics

Of the 1240 analyzed patients, 64 met the eligibility criteria. 30 (46.9%) and 34 (53.1%) patients were included in the PD and Without PD groups, respectively (Figure 1B). Twenty‐eight (82.4%) and six (17.6%) patients in the Without PD group discontinued the initial ICI treatment owing to irAEs and clinical decisions, respectively. The median follow‐up period for censored cases was 34.5 months in the overall population. The patients characteristics at the initiation of ICI retreatment are listed in Table 1. Most patients had ECOG‐PS scores of 0 or 1, and for 60% of them retreated ICI after the third line of their treatment course. No significant differences were observed between the PD and Without PD groups in terms of age, sex, clinical stage, or histological subtypes. The percent of patients with a PD‐L1 TPS ≥50% was significantly higher in the Without PD group than in the PD group (Without PD group vs. PD group: 61.8% vs. 26.7%, *p* = 0.006).

**TABLE 1:** Patient characteristics.

Factors	Overall (*n* = 64)	PD group (*n* = 30)	Without PD group (*n* = 34)
Age, median (range)	67 (38–87)	64.5 (38–86)	68 (44–87)
Sex, *n* (%)			
Male	46 (71.9)	20 (66.7)	26 (76.5)
Female	18 (28.1)	10 (33.3)	8 (23.5)
ECOG‐PS, *n* (%)			
0	13 (20.3)	5 (16.7)	8 (23.5)
1	44 (68.8)	22 (73.3)	22 (64.7)
≥2	7 (10.9)	3 (10.0)	4 (11.8)
Histology, *n* (%)			
Sq	19 (29.7)	9 (30.0)	10 (29.4)
Non‐Sq	53 (82.8)	21 (70.0)	32 (94.1)
Smoking status, *n* (%)			
Never	29 (45.3)	23 (76.7)	6 (17.6)
Former/current	35 (54.7)	7 (23.3)	28 (82.4)
Clinical stage, *n* (%)			
III–IV	39 (60.9)	17 (56.7)	22 (64.7)
Recurrence	25 (39.1)	13 (43.3)	12 (35.3)
*EGFR* mutation, *n* (%)			
Positive	4 (6.3)	3 (10.0)	1 (2.9)
Negative	60 (93.8)	27 (90.0)	33 (97.1)
PD‐L1 expression, *n* (%)			
≥50%	29 (45.3)	8 (26.7)	21 (61.8)
1%–49%	15 (23.4)	8 (26.7)	7 (20.6)
<1%	11 (17.2)	9 (30.0)	2 (5.9)
Not evaluated	9 (14.1)	5 (16.7)	4 (11.8)
Treatment line of ICI rechallenge, *n* (%)			
2	22 (34.4)	6 (20.0)	16 (47.1)
≥3	42 (65.6)	24 (80.0)	18 (52.9)
Period for interval, median (month, range)	8.5 (0.7–43.5)	5.9 (0.7–19.5)	10.4 (2.6–43.5)
Number of chemo during interval, *n* (%)			
0	41 (64.1)	13 (43.3)	28 (82.4)
1	15 (23.4)	10 (33.3)	5 (14.7)
≥2	8 (12.5)	7 (23.3)	1 (2.9)
Radiation during interval, *n* (%)			
Yes	13 (20.6)	9 (30.0)	4 (11.7)
No	50 (79.4)	21 (70.0)	30 (88.3)
Objective response rate in initial ICI, *n* (%)	35 (54.7)	10 (33.3)	25 (73.5)
Disease control rate in initial ICI, *n* (%)	52 (81.3)	20 (66.7)	32 (94.1)
Best overall response in initial ICI, *n* (%)			
CR	1 (1.6)	0 (0.0)	1 (2.9)
PR	34 (53.1)	10 (33.3)	24 (70.6)
SD	17 (26.6)	10 (33.3)	7 (20.6)
PD	0 (0.0)	0 (0.0)	0 (0.0)
Non CR/non PD	12 (18.8)	10 (33.3)	2 (5.9)
NE	0 (0.0)	0 (0.0)	0(0.0)

Abbreviations: CR, complete response; ECOG‐PS, Eastern Cooperative Oncology Group performance status; EGFR, epidermal growth factor receptor; ICI, immune checkpoint inhibitor; Ipi, ipilimumab; irAE, immune‐related adverse event; NE, not evaluated; Nivo, nivolumab; PD, progressive disease; PD‐1, programmed death −1, PD‐L1, programmed death ligand‐1; PR, partial response; SD, stable disease; Sq, squamous cell carcinoma.

Detailed data on initial ICI treatment are shown in Table S1. In total, 12.5% of the patients received their initial ICI in combination with chemotherapy (e.g., pembrolizumab plus carboplatin‐pemetrexed). The median treatment duration of the initial ICI was 5.5 months (range 0.7–25.4 months) in the PD group and 2.6 months (range 0–23.2 months) in the Without PD group, respectively. In the overall population, the ORR and DCR of the initial ICI treatment were 54.7% and 81.3%, respectively. The ORR of the initial ICI treatment was significantly higher in the Without PD group than in the rechallenge group (Without PD group vs. PD group: 73.5% vs. 33.3%, *p* < 0.01).

ICI retreatment regimens are shown in Figure 1B. In 24 patients, one type of ICI was switched to another type of ICI: from anti‐PD‐1 (e.g. nivolumab, pembrolizumab) to PD‐L1 (e.g. atezolizumab) or vice versa. Switching administration was more frequent in the PD group than in Without PD group (PD vs. Without PD group: 63.3% vs. 14.7%, *p* < 0.01). Additionally, four patients in the PD group (Figure S1A; #5, #14, #20, #23) received as a second course of ICI therapy, a combination of nivolumab and ipilimumab.

### 3.2 Efficacy of ICI retreatment in PD and Without PD group

In the overall population, the ORR and DCR for the ICI retreatment were 18.8% and 37.5%, respectively (Table 2). The ORR in the Without PD group was significantly higher than that in the PD group (PD vs. Without PD: 6.7% vs. 29.4%; *p* = 0.03).

**TABLE 2:** Tumor response in ICI retreatment.

	ICI retreatment		
	Overall	PD group	Without PD group
	*N* = 64	*N* = 30	*N* = 34
Objective response rate—*n* (%)	12 (18.8)	2 (6.7)	10 (29.4)
Disease control rate—*n* (%)	24 (37.5)	7 (23.3)	17 (50.0)
Best overall response—*n* (%)			
CR	2 (3.1)	0 (0.0)	2 (5.9)
PR	10 (15.6)	2 (6.7)	8 (23.5)
SD	12 (18.8)	5 (16.7)	7 (20.6)
PD	29 (45.3)	14 (46.7)	15 (44.1)
Non‐CR, non‐PD	6 (9.4)	5 (16.7)	1 (2.9)
NE	5 (7.8)	4 (13.3)	1 (2.9)

Abbreviations: CI, confidence interval; CR, complete response; ICI, immune checkpoint inhibitor; NE, not evaluated; PD, progressive disease; PR, partial response; SD, stable disease.

**FIGURE 2:**
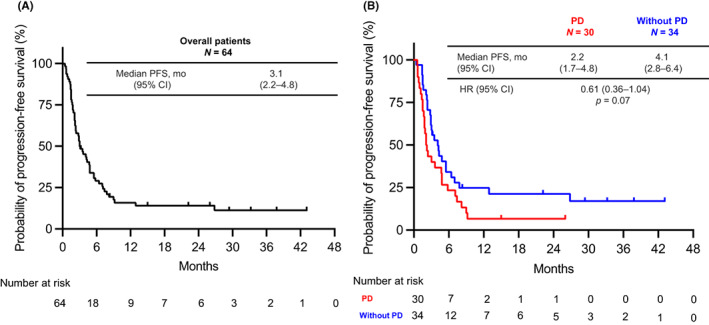
Progression‐free survival (PFS) for ICI retreatment (overall patient). (B) PFS for ICI retreatment (PD vs. Without PD). HR, hazard ratio; CI, immune checkpoint inhibitor; mo, months; PD, progressive disease.

The PFS‐R in the overall population was 3.1 months (95% CI, 2.2–4.8 months) (Figure 2A). The PFS in the Without PD group was longer than that of the PD group (PD vs. Without PD group: 2.2 months [95% CI, 1.7–4.8 months] vs. 4.1 months [95% CI, 2.8–6.4 months]; HR, 0.61; 95% CI, 0.36–1.04; *p* = 0.07) (Figure 2B), although the difference was not statistically significant. However, multivariate Cox regression analysis showed the definition of ICI re‐administration as an independent factor affecting PFS (Without PD group vs. PD group, HR 0.42, 95% CI, 0.21–0.85; *p* = 0.015), suggesting a benefit of ICI re‐administration in the Without PD group (Table 3).

**TABLE 3:** Univariate and multivariate Cox regression analysis for PFS prediction.

Characteristics	Risk factors	Univariate			Multivariate		
		HR	95% CI	*p*‐Value	HR	95% CI	*p*‐Value
Age	≥75 year (vs. <75 year)	1.28	0.63–2.56	0.49	1.31	0.60–2.83	0.49
Sex	Male (vs. female)	0.89	0.50–1.59	0.69	1.33	0.40–4.46	0.65
Smoking history	Current/ex‐smoker (vs. never smoker)	0.57	0.30–1.08	0.08	0.39	0.11–1.39	0.15
Histology	Non‐Sq (vs. Sq)	1.35	0.75–2.43	0.32	1.59	0.77–3.29	0.21
PD‐L1 expression	≥1% (vs. <1%)	1.16	0.57–2.34	0.69	2.03	0.83–4.98	0.12
Chemotherapy during interval	Yes (vs. no)	0.69	0.40–1.19	0.18	0.67	0.34–1.32	0.25
Retreatment definition	Without PD group (vs. PD group)	0.61	0.36–1.04	0.07	0.42	0.21–0.85	0.015*

Abbreviations: CI, confidence interval; HR, hazard ratio; PD, progressive disease; PD‐L1, programmed death ligand‐1; Sq, Squamous cell carcinoma.

* *p* < 0.05.

**FIGURE 3:**
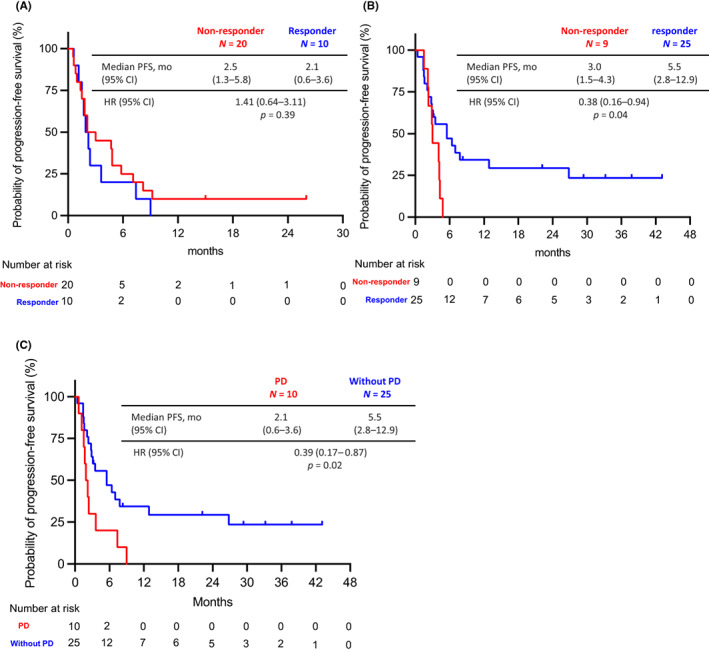
(A) Progression‐free survival (PFS) for ICI retreatment according to initial ICI response in PD group and (B) Without PD group. (C) PFS in retreatment (PFS‐R) between the PD and Without PD groups in the responder patients of the initial ICI treatment. CI, confidence interval; HR, hazard ratio; ICI, immune checkpoint inhibitor; mo, months; PD, progressive disease.

Within PD and Without PD groups, patients were classified into responders (patients with CR or PR) or non‐responders (patients with SD or non‐CR/non‐PD [patients without target lesions]) based on the response to the initial ICI treatment. In the PD group, PFS‐R was not significantly different between non‐responder and responder patients (non‐responder vs. responder: median PFS‐R 2.5 months [95% CI, 1.3–5.8 months] vs. 2.1 months [95% CI, 0.6–3.6 months]; HR, 1.41; 95% CI, 0.64–3.11; *p* = 0.39) (Figure 3A). In contrast, in the Without PD group, PFS‐R was significantly longer in the responder than in the non‐responder patients (non‐responder vs. responder: median PFS‐R 3.0 months [95% CI, 1.5–4.3 months] vs. 5.5 months [95% CI, 2.8–12.9 months]; HR, 0.38; 95% CI, 0.16–0.96; *p* = 0.04) (Figure 3B). We next compared the PFS‐R between the PD and Without PD groups in the responder patients of the initial ICI treatment. PFS‐R was significantly longer in the Without PD group than in the PD group (PD vs. Without PD group: median PFS‐R 2.1 months [95% CI, 0.6–3.6 months] vs. 5.5 months [95% CI, 2.8–12.9 months]; HR, 0.39; 95% CI, 0.17–0.87; *p* = 0.02) (Figure 3C).

Regarding the durable response to the retreatment with ICI (PFS‐R ≥ 2 years), no patients in the PD group experienced a durable response, while five patients (14.7%) in the Without PD group did. The latter presented only mediastinal lymph node progression during the treatment discontinuation period, after the initial ICI therapy (patients #1 to #5 in Figure S1B).

### 3.3 Switching ICI regimen as retreatment strategy

We evaluated whether switching ICIs (from anti‐PD‐1 to anti‐PD‐L1 or vice versa) affected clinical outcomes. No significant differences in PFS‐R were observed between the switching and non‐switching treatment groups, in either the PD or Without PD group (non‐switching vs. switching PD groups: median PFS, 3.6 months [95% CI, 1.5–7.4 months] vs. 2.1 months [95% CI, 1.2–4.8 months], HR 1.33; 95% CI, 0.61–2.89; *p* = 0.47; non‐switching vs. switching PD groups; median PFS, 4.2 months [95% CI, 2.9–7.0 months] vs. 2.2 months [95% CI, 1.4 months to not reached], HR 1.36; 95% CI, 0.47–3.96; *p* = 0.57) (Figure S2A,B).

Next, we investigated the clinical outcomes of the retreatment with the combination nivolumab plus ipilimumab. Out of the four patients in the PD group who received nivolumab and ipilimumab, no patient responded to the combination therapy. The median PFS‐R in this subgroup was 2.1 months (95% CI, 1.5 months–not reached).

### 3.4 The difference in clinical outcomes between initial ICI therapy and retreatment

**FIGURE 4:**
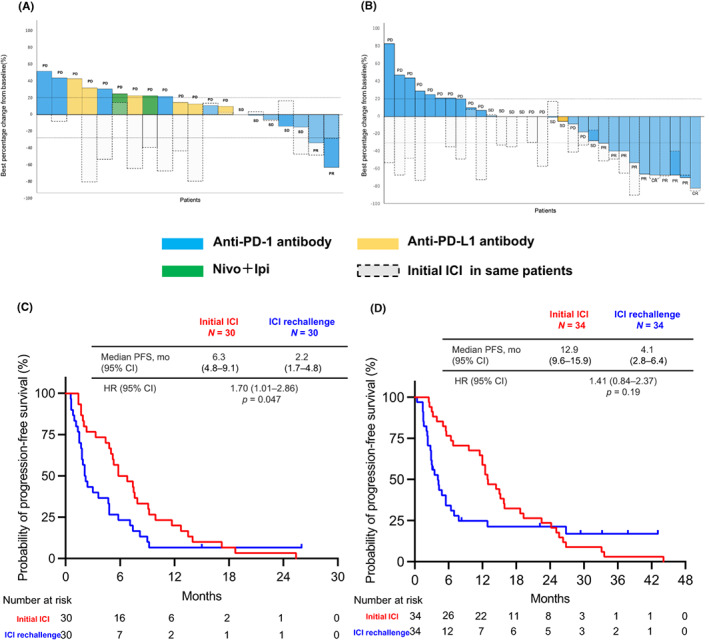
(A) Waterfall plot of initial and second ICI in PD group. (B) Waterfall plot of initial and retreatment ICI in Without PD group. The colored bars (blue, yellow, green) represent the response of the retreatment ICI, and the gray dashed bars represent the response of the initial ICI in the same patient. (C) PFS in PD group (Initial ICI vs. ICI rechallenge). (D) PFS in Without PD group (Initial ICI vs. ICI rechallenge). CI, confidence interval; HR, hazard ratio; ICI, immune checkpoint inhibitor; Ipi, ipilimumab; mo, months; Nivo, nivolumab; PD‐1, programmed death 1; PFS, progression‐free survival.

We compared the ORR and PFS of initial ICI therapy with those of the retreatment (Figure 4A,B). The percentage of CR or PR patients who responded to both the initial ICI and retreatment was higher in the Without PD group than in the PD group (Without PD vs. PD: 29.4% vs. 3.3%, *p* = <0.01).

The median PFS in the initial ICI treatment (PFS1) group was significantly longer than the median PFS‐R in the PD group (median PFS1 vs. median PFS‐R: 6.3 months [95% CI, 4.8–9.1 months] vs. 2.2 months [95% CI, 1.7–4.8 months]; HR, 1.70; 95% CI, 1.01–2.86; *p* = 0.047) (Figure 4C). Similar results were observed in the Without PD group (median PFS1 vs. median PFS‐R: 12.9 months [95% CI, 9.6–15.9 months] vs. 4.1 months [95% CI, 2.8–6.4 months]; HR, 1.41; 95% CI, 0.84–2.37; *p* = 0.19) (Figure 4D).

### 3.5 Safety of initial ICI therapy and retreatment

The irAEs of the ICIs administered as initial therapy or retreatment are shown in Table 4. The incidence of the irAEs during initial ICI therapy was 71.9% and 18.8% for all‐grade irAE and grade 3 or higher, respectively. Of the 64 patients who developed irAEs during the initial ICI therapy, 29 (45.3%) required systemic corticosteroids and 29 (45.3%) discontinued the therapy.

**TABLE 4:** Immune‐related adverse events during initial and ICI retreatment.

irAEs	irAEs during initial ICI *n* = 64					irAEs during ICI retreatment *n* = 64				
	Total	Gr1/2	Gr3/4	Corticosteroids	Leading to discontinue	Total	Gr1/2	Gr3/4	Corticosteroids	Leading to discontinue
Any irAEs, *n* (%)	46 (71.9)	34 (53.1)	12 (18.8)	29 (45.3)	29 (45.3)	18 (28.1)	13 (20.3)	5 (7.8)	13 (20.3)	11 (17.2)
Pneumonitis, *n* (%)	15 (23.4)	13 (20.3)	2 (3.1)	13 (20.3)	12 (18.8)	8 (12.5)	6 (9.4)	2 (3.1)	7 (10.9)	7 (10.9)
Colitis, *n* (%)	11 (17.2)	8 (12.5)	3 (4.7)	7 (10.9)	6 (9.4)	5 (7.8)	2 (3.1)	3 (4.7)	6 (9.4)	4 (6.3)
Hypo/hyper thyroidism, *n* (%)	10 (15.6)	10 (15.6)	0 (0.0)	0 (0.0)	2 (3.1)	3 (4.7)	3 (4.7)	0 (0.0)	0 (0.0)	0 (0.0)
Rash, *n* (%)	10 (15.6)	10 (15.6)	0 0.0	1 (1.6)	1 (1.6)	2 (3.1)	2 (3.1)	0 (0.0)	0 (0.0)	0 (0.0)
Hepatitis, *n* (%)	4 (6.3)	2 (3.1)	2 (3.1)	2 (3.1)	2 (3.1)	0 (0.0)	0 (0.0)	0 (0.0)	0 (0.0)	0 (0.0)
Adrenal insufficiency, *n* (%)	3 (4.7)	2 (3.1)	1 (1.6)	3 (4.7)	1 (1.6)	0 (0.0)	0 (0.0)	0 (0.0)	0 (0.0)	0 (0.0)
Arthritis, *n* (%)	2 (3.1)	0 (0.0)	2 (3.1)	2 (3.1)	2 (3.1)	0 (0.0)	0 (0.0)	0 (0.0)	0 (0.0)	0 (0.0)
Encephalitis, *n* (%)	1 (1.6)	0 (0.0)	1 (1.6)	1 (1.6)	1 (1.6)	0 (0.0)	0 (0.0)	0 (0.0)	0 (0.0)	0 (0.0)
Pericarditis, *n* (%)	1 (1.6)	0 (0.0)	1 (1.6)	1 (1.6)	1 (1.6)	0 (0.0)	0 (0.0)	0 (0.0)	0 (0.0)	0 (0.0)
Infusion reaction, *n* (%)	1 (1.6)	1 (1.6)	0 (0.0)	0 (0.0)	0 (0.0)	1 (1.6)	1 (1.6)	0 (0.0)	0 (0.0)	0 (0.0)
AMY elevation, *n* (%)	1 (1.6)	1 (1.6)	0 (0.0)	0 (0.0)	1 (1.6)	0 (0.0)	0 (0.0)	0 (0.0)	0 (0.0)	0 (0.0)
Diabetes mellitus, *n* (%)	1 (1.6)	0 (0.0)	1 (1.6)	0 (0.0)	0 (0.0)	0 (0.0)	0 (0.0)	0 (0.0)	0 (0.0)	0 (0.0)

Abbreviations: Gr, Grade; ICI, immune checkpoint inhibitor; irAEs, immune‐related adverse events.

During ICI retreatment, 18 of 64 patients (28.1%) developed irAEs. Grade 3 or higher irAEs were observed in 5 of the 64 patients (7.8%). 11 of the 64 patients (17.2%) discontinued treatment due to irAEs after ICI retreatment. The median number of days from the first administration of the ICI until the onset of an irAE requiring discontinuation was 49.5 days (range: 3–396 days) for the initial ICI and 46 days (range: 1–58 days) ICI rechallenge, respectively.

Of the 28 patients who discontinued their initial ICI treatment due to irAEs, 10 (35.7%) developed irAEs during ICI retreatment, including eight (28.6%) experiencing the same irAEs as the initial irAE (“relapse”) (Table S2). Among the 12 patients who experienced grade 3 or higher irAEs during initial ICI treatment, 8 did not develop a recurrence of irAEs during retreatment, whereas four developed irAEs during ICI retreatment (grade 1: one patient [hypothyroidism], grade 2: two patients [colitis, infusion reaction], and grade 3: one patient [colitis], #1, #16, #17, #31 in Figure S1B).

Finally, we performed a univariate analysis to evaluate the risk factors for irAE recurrence in patients who discontinued their initial ICI treatment due to irAE (*n* = 28). The results showed that clinical factors, such as the grade of initial irAEs and interval period, were not significantly associated with recurrent irAE (Table 5).

**TABLE 5:** Univariate analysis regarding irAEs during ICI retreatment for patients with a previous history of ICI discontinue due to irAEs.

Factors	No. of patients	No. of events	Univariable		*p*‐Value
			HR	(95% CI)	
Age			0.95	(0.88–1.03)	0.24
Sex					1.00
Female	7	3	Reference		
Male	21	9	1.00	(0.18–5.63)	
ECOG‐PS					0.19
0–1	24	9	Reference		
≥2	4	3	5.00	(0.45–55.60)	
Smoking					0.60
Never	6	2	Reference		
Former, current	22	10	1.67	(0.25–11.10)	
PD‐L1 expression					0.82
<50%	11	5	Reference		
≥50%	17	7	0.84	(0.18–3.88)	
Duration of interval			0.95	(0.87–1.04)	0.26
irAE grade during initial ICI					0.38
Grade 1/2	16	8	Reference		
Grade 3/4	12	4	0.5	(0.11–2.35)	

Abbreviations: CI, confidence interval; ECOG‐PS, Eastern Cooperative Oncology Group performance status; ICI, immune checkpoint inhibitor; irAEs, immune‐related adverse events; PD‐L1, programmed death ligand‐1; PS performance status.

## 4 DISCUSSION

In this study, we investigated the efficacy and safety of ICI retreatment in patients with advanced NSCLC and found that disease status at the time of initial ICI discontinuation affected the efficacy of ICI retreatment. The efficacy of ICI retreatment was limited when ICI treatment was discontinued due to disease progression (“PD group”). In contrast, ICI re‐administration to patients without disease progression at the time of initial ICI discontinuation (“Without PD group”) showed better efficacy than that seen in the PD group, especially in patients who responded to initial ICI therapy.

The reported clinical outcomes of ICI retreatment in advanced NSCLC patients are varied: ORR ranged from 0 to 43% and PFS from 1.5 to 15.4 months.^2^, ^3^, ^4^, ^5^, ^6^, ^7^, ^8^, ^9^, ^10^, ^11^, ^12^ However, a meta‐analysis reported an ORR of 12.4% and a median PFS of 3.7 months after ICI retreatment.^19^ Our study showed that the ORR and median PFS of ICI retreatment in the overall population were 18.8% and 3.1 months, respectively, which was comparable to previous reports. Additionally, Gobbini et al. reported that patients who discontinued their initial ICI treatment owing to irAEs or clinical decisions exhibited better outcomes with ICI retreatment than those who discontinued initial therapy owing to disease progression.^6^ In the KEYNOTE‐010 trial^20^ comparing pembrolizumab versus docetaxel in previously treated advanced NSCLC patients, among 21 patients who progressed after the completion of the 2‐year treatment plan without disease progression, and resumed pembrolizumab, more than half of the patients (52.4%) showed a partial response or stable disease. These studies suggest an association between the reason for discontinuation of initial ICI and the ICI retreatment efficacy. Indeed, our study showed that disease status at the time of initial ICI discontinuation affected ICI readministration efficacy, because Without PD group patients had significantly better clinical outcomes than the PD group, and the PD group was not effective regardless of the initial ICI efficacy.

Furthermore, long‐term responders (≥2 years PFS‐R) were observed in the Without PD group. All these patients presented with only progression of lymph node metastases during the treatment discontinuation period after the initial ICI therapy, suggesting these patients are good candidates for ICI retreatment.

Regarding ICI retreatment regimens, in our study, 42.3% of patients switched from an anti‐PD‐1 to anti‐PD‐L1 antibody or vice versa. The clinical significance of this switch is not fully understood. Kitagawa et al.^8^ reported that ICI retreatment based on switching administration of anti‐PD‐1 and anti‐PD‐L1 antibodies in NSCLC patients has a median PFS of 4.0 months. In this study, there were no significant differences in PFS‐R between the switching and non‐switching treatment subgroups in either the PD or Without PD groups. Additionally, some patients switched from an initial anti‐PD‐1/anti‐PD‐L1 antibody to ICIs combination therapy (nivolumab plus ipilimumab) for retreatment in the PD group. In patients with renal cell carcinoma, salvage therapy with nivolumab and ipilimumab after prior anti‐PD‐1/PD‐L1 failure had limited efficacy with a 20% ORR and a median PFS of 4.0 months.^21^ In our study, the combination of nivolumab and ipilimumab as a retreatment ICI therapy had limited efficacy (ORR, 0%; median PFS, 2.1 months). Our results suggest that once prior therapy targeting the anti‐PD‐1/PD‐L1 pathway has failed, it is difficult to activate immune function even when combined with anti‐CTLA‐4 antibodies. Based on our results, ICI retreatment is not recommended for patients showing resistance during ICI treatment regardless of the type of ICI regimen.

A meta‐analysis of studies in which ICI therapy was readministered in patients with solid tumors following initial discontinuation due to irAE found that the irAE recurrence rate was 34.2%.^22^ In addition, 28.8% of patients who received ICI retreatment developed the same irAEs as patients with solid tumors.^22^ In the study, re‐administration of ICI to patients who had discontinued initial ICI therapy due to irAEs resulted in 35.7% of patients experiencing irAEs, specifically 28.6% of patients experiencing a recurrence of the same irAE. In contrast, previous studies indicated no association between the severity of the initial irAE and the recurrence rate of irAE,^23^, ^24^ similar to the findings of the present study. However, these findings do not warrant a definitive conclusion that ICI can be safely re‐administered to patients with a history of grade 3 or higher irAEs during initial ICI therapy. Therefore, ICI retreatment in patients who have developed irAEs should be approached with caution and based on various factors such as the type and severity of toxicity, time to resolution of toxicity, and previous tumor response.

This study has several limitations. First, it is a small sample‐sized, single‐center, retrospective study with bias in the selection of ICI treatment regimens and timing of radiological assessments. However, most previous studies examining the efficacy and safety of ICI retreatment also had similar sample sizes to our study. Furthermore, previous data on ICI retreatment included patients who discontinued ICI due to irAEs rather than disease progression events and resumed ICI before developing disease progression.^5^, ^7^, ^11^, ^12^ Thus, our data suggest that ICI retreatment may be beneficial for patients who discontinued ICI therapy for reasons other than disease progression and experienced cancer progression during the withdrawal period, but not for those who experienced disease progression during initial ICI treatment. Further research with larger patient numbers is needed to confirm our findings and determine the distinct clinical outcomes of ICI retreatment according to disease progression status during initial ICI treatment. Second, this study included patients who experienced grade ≥3 irAEs during initial ICI treatment. Permanent discontinuation of treatment is generally recommended after the onset of grade ≥3 irAEs.^13^ In this study, there was a potential bias in patient selection for ICI retreatment. Thus, it is difficult to conclude that readministering ICI after severe irAEs during initial ICIs is safe. Given the benefits of ICIs, it is necessary to further examine the recurrent risk factors for irAEs and which patients can undergo ICI retreatment safely, after experiencing severe irAEs in the initial ICI therapy.

## 5 CONCLUSION

ICI retreatment showed efficacy in patients who discontinued ICI therapy for reasons other than disease progression and subsequently experienced disease progression during the withdrawal period. However, ICI retreatment was ineffective in patients with disease progression during the initial ICI treatment.

## AUTHOR CONTRIBUTIONS

**Masahiro Torasawa:** Conceptualization (equal); data curation (lead); formal analysis (lead); investigation (lead); methodology (lead); project administration (equal); resources (lead); visualization (lead); writing – original draft (lead); writing – review and editing (lead). **Tatsuya Yoshida:** Conceptualization (equal); data curation (supporting); formal analysis (supporting); investigation (supporting); methodology (supporting); project administration (equal); resources (supporting); supervision (lead); visualization (supporting); writing – original draft (supporting); writing – review and editing (lead). **Yuki Takeyasu:** Investigation (supporting); writing – review and editing (supporting). **Yukiko Shimoda:** Resources (supporting); writing – review and editing (supporting). **Akiko Tateishi:** Resources (supporting); writing – review and editing (supporting). **Yuji Matsumoto:** Resources (supporting); writing – review and editing (supporting). **Ken Masuda:** Resources (supporting); writing – review and editing (supporting). **Yuki Shinno:** Resources (supporting); writing – review and editing (supporting). **Yusuke Okuma:** Resources (supporting); writing – review and editing (supporting). **Yasushi Goto:** Resources (supporting); writing – review and editing (supporting). **Hidehito Horinouchi:** Resources (supporting); writing – review and editing (supporting). **Noboru Yamamoto:** Resources (supporting); writing – review and editing (supporting). **Kazuhisa Takahashi:** Supervision (supporting); writing – review and editing (supporting). **Yuichiro Ohe:** Resources (supporting); supervision (supporting); writing – review and editing (supporting).

## FUNDING INFORMATION

This research did not receive any specific grant from funding agencies in the public, commercial, or not‐for‐profit sectors.

## CONFLICT OF INTEREST STATEMENT

MT has nothing to disclose. TY reports grants from ONO Pharmaceutical, grants and personal fees from Bristol‐Myers Squibb, personal fees from Chugai, grants and personal fees from AstraZeneca, grants from MSD, during the conduct of the study; grants from Takeda, personal fees from Novartis, grants from Abbvie, outside the submitted work. YT has nothing to disclose. YS has nothing to disclose. AT has nothing to disclose. YM reports personal fees from AstraZeneca, during the conduct of the study; grants from National Cancer Center Research and Development Fund, grants from Grant‐in‐Aid for Scientific Research on Innovative Areas, grants from Hitachi, Ltd., grants from Hitachi High‐Technologies, personal fees from Olympus, personal fees from Novartis, personal fees from COOK, personal fees from AMCO INC. outside the submitted work. KM reports personal fees from Chugai, personal fees from AstraZeneca, during the conduct of the study. YS reports grants from ONO Pharmaceutical, personal fees from AstraZeneca, personal fees from Chugai Pharmaceutical, during the conduct of the study; personal fees from Pfizer, grants from Japan Clinical Research Operations K.K., grants from Janssen Pharmaceutical K.K., outside the submitted work; YO reports grants from AbbVie, outside the submitted work. YG reports grants and personal fees from Ono Pharmaceutical, grants, and personal fees from MSD, grants and personal fees from Bristol Myers Squibb, personal fees from Chugai, personal fees from AstraZeneca, during the conduct of the study; grants and personal fees from Eli Lilly, grants and personal fees from Taiho Pharmaceutical, personal fees from Boehringer Ingelheim, grants and personal fees from Pfizer, grants and personal fees from Novartis, grants and personal fees from Guardant Health, grants from Kyorin, grants and personal fees from Dai‐ichi Sankyo, personal fees from Illumina, outside the submitted work. HH reports grants and personal fees from MSD, grants and personal fees from Ono Pharmaceutical, grants and personal fees from Bristol‐Myers Squibb S, grants and personal fees from Chugai, grants and personal fees from Astra Zeneca, during the conduct of the study; grants and personal fees from Taiho, grants from Astellas, grants from Merck Serono, grants from Genomic Health, grants and personal fees from Lilly, outside the submitted work. NY reports grants and personal fees from Bristol‐Myers Squibb, grants from MSD, grants and personal fees from Ono Pharmaceutical, grants and personal fees from Chugai, personal fees from AstraZeneca, during the conduct of the study; grants from Taiho, grants and personal fees from Eisai, grants and personal fees from Lilly, grants from Quintiles, grants from Astellas, grants from Novartis, grants from Daiichi‐Sankyo, grants and personal fees from Pfizer, grants from Boehringer Ingelheim, grants from Kyowa‐Hakko Kirin, grants from Bayer, grants and personal fees from Takeda, grants and personal fees from Otsuka, personal fees from Boehringer Ingelheim, personal fees from Cimic, grants from Janssen Pharma, grants from Merck, personal fees from Sysmex, grants from GSK, grants from Sumitomo Dainippon, grants from Shionogi, outside the submitted work. KT reports grants and personal fees from ONO Pharmaceutical, personal fees from Bristol‐Myers Squibb, grants and personal fees from MSD, grants and personal fees from Chugai Pharmaceutical, personal fees from AstraZeneca, during the conduct of the study; grants and personal fees from Nippon Boehringer Ingelheim, grants from Glaxo SmithKline Consumer Healthcare Japan, grants from NIPPON SHINYAKU, grants from TSUMURA & CO., grants and personal fees from Pfizer Inc., grants and personal fees from TAIHO PHARMACEUTICAL, grants from DAIICHI SANKYO, grants from Astellas Pharma Inc., grants and personal fees from KYORIN Pharmaceutical, grants from KYOWA Hakko Kirin, grants from TEIJIN PHARMA LIMITED, grants from Sanofi, grants from Shionogi, grants and personal fees from Novartis Pharma, grants and personal fees from Eli Lilly Japan, grants from Actelion Pharmaceuticals Japan Ltd., grants from NIPRO PHARMA CORPORATION, grants from Takeda Pharmaceutical Company Limited., grants from Bayer Yakuhin, grants from Torii Pharmaceutical, personal fees from Meiji Seika Pharma, outside the submitted work. YO reports grants and personal fees from Bristol‐Myers Squibb, grants and personal fees from ONO Pharmaceutical, grants and personal fees from MSD, grants and personal fees from AstraZeneca, grants and personal fees from Chugai, during the conduct of the study; personal fees from Boehringer Ingelheim, personal fees from Celtrion, grants and personal fees from Eli Lilly, grants and personal fees from Janssen, grants and personal fees from Kyorin, grants from Kissei, grants and personal fees from Nippon Kayaku, grants and personal fees from Novartis, grants and personal fees from Pfizer, grants and personal fees from Taiho, grants and personal fees from Takeda Pharmaceutical, outside the submitted work.

## ETHICS STATEMENT

The present study with human samples has been approved by the Ethics Committee of the National Cancer Center Hospital, Tokyo, Japan (2015‐355).

## Supporting information


Figure S1.

Table S1.

## Data Availability

Data are available upon reasonable request.
