# Supplementary material for: Disease progression status during initial immune checkpoint inhibitor (ICI) affects the clinical outcome of ICI retreatment in advanced non‐small cell lung cancer patients
Source: Cancer Med. 2023 Apr 16;12(11):12388–401. doi: 10.1002/cam4.5939 (PMC10278515; doi:10.1002/cam4.5939)
Supplement: Supplementary file 1 — Figure S1. [file CAM4-12-12388-s002.pptx]

## Slide 1
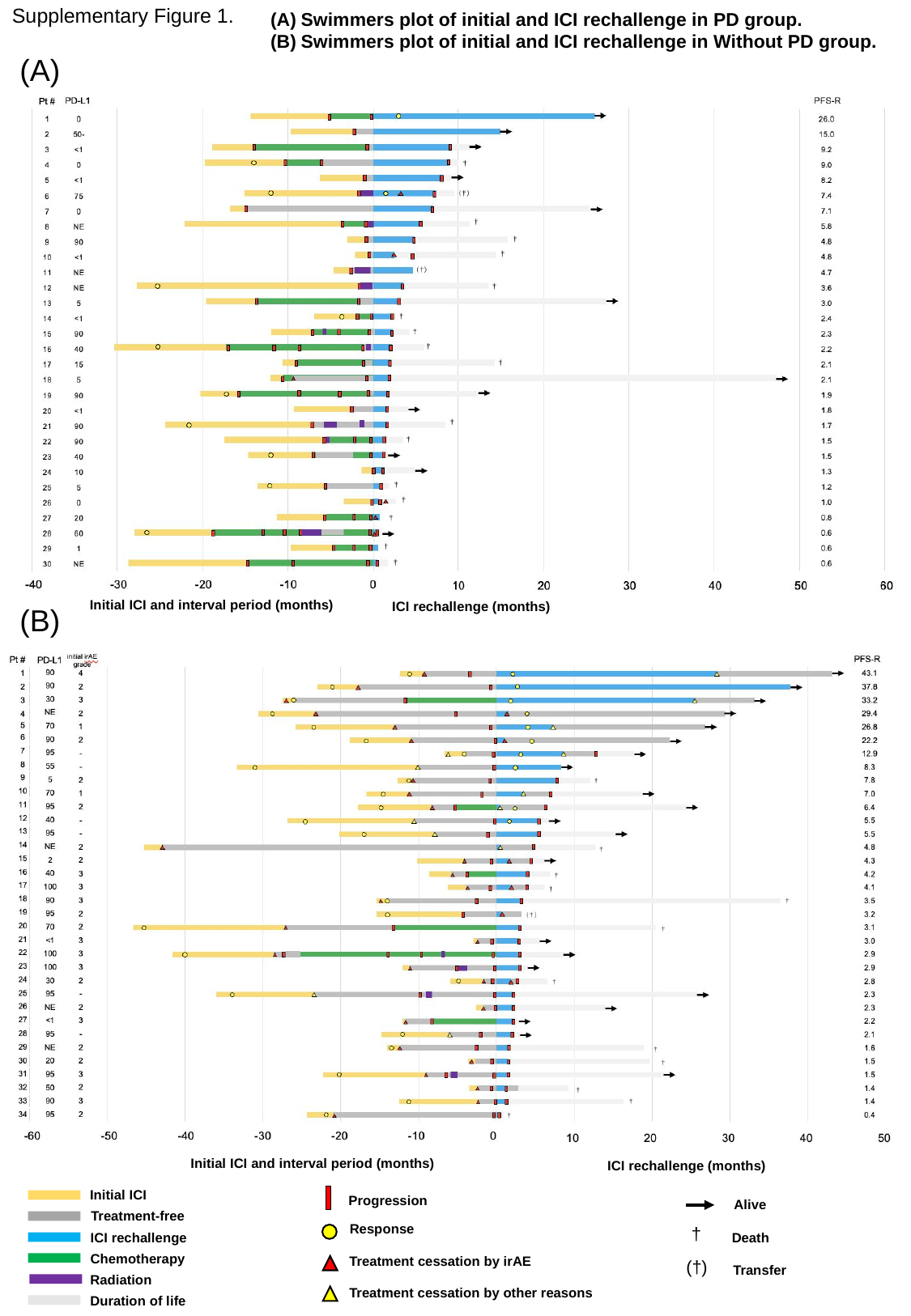

Supplementary Figure 1.
Swimmers plot of initial and ICI rechallenge in PD group.
Swimmers plot of initial and ICI rechallenge in Without PD group.
(A)
Initial ICI and interval period (months)
ICI rechallenge (months)
(B)
Initial ICI and interval period (months)
ICI rechallenge (months)
Initial ICI
Progression
Alive
Treatment-free
Response
†
ICI rechallenge
Death
Chemotherapy
Treatment cessation by irAE
(†)
Transfer
Radiation
Treatment cessation by other reasons
Duration of life

## Slide 2
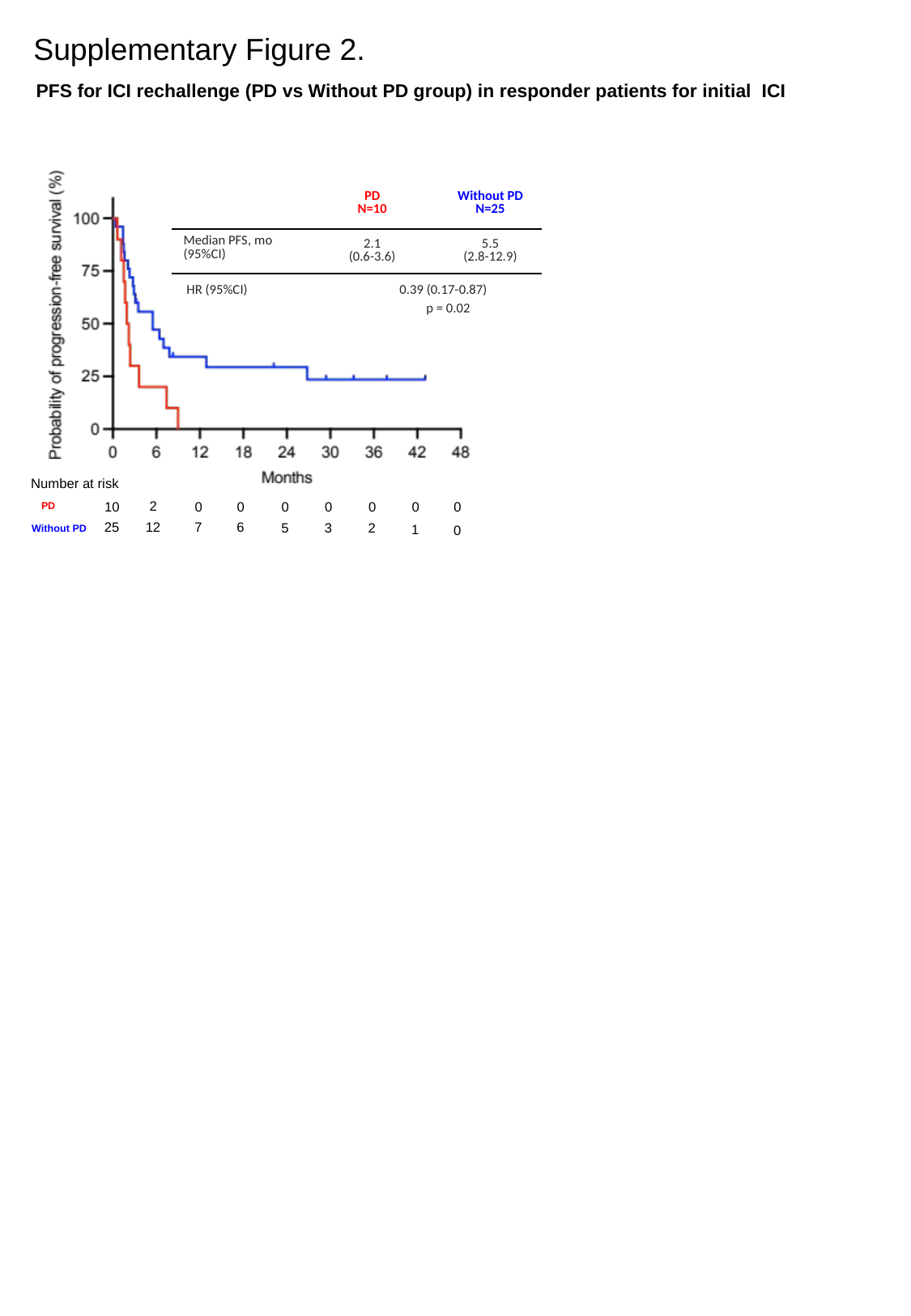

Supplementary Figure 2.
PFS for ICI rechallenge (PD vs Without PD group) in responder patients for initial ICI
| | PD N=10 | Without PD N=25 |
| --- | --- | --- |
| Median PFS, mo (95%CI) | 2.1 (0.6-3.6) | 5.5 (2.8-12.9) |
| HR (95%CI) 　　　　 　　　　　0.39 (0.17-0.87) 　　 　　　　　 　　　　 p = 0.02 | | |
Number at risk
2
10
0
0
0
0
0
0
0
PD
25
12
7
6
5
3
2
1
0
Without PD

## Slide 3
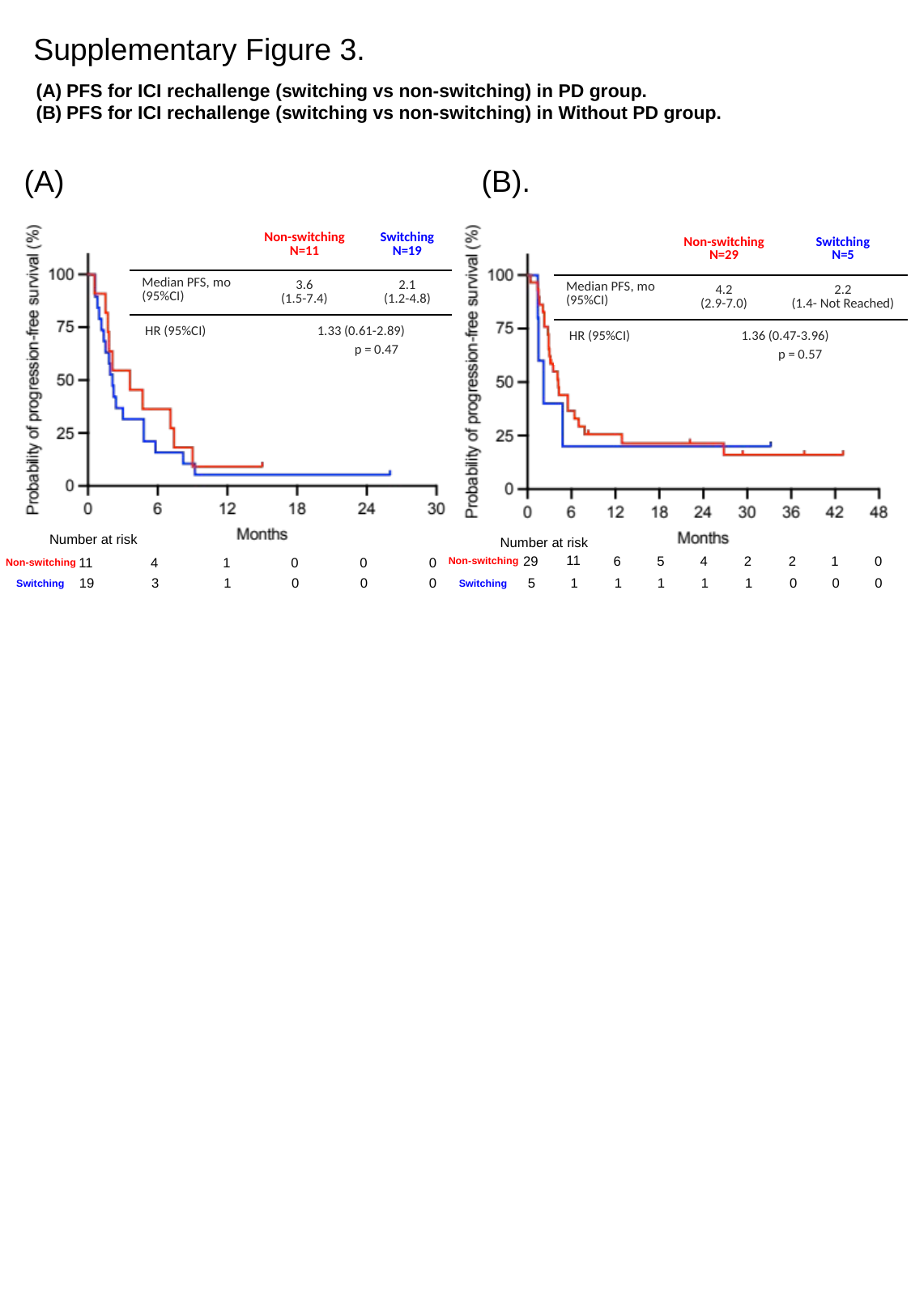

Supplementary Figure 3.
PFS for ICI rechallenge (switching vs non-switching) in PD group.
PFS for ICI rechallenge (switching vs non-switching) in Without PD group.
(A)
(B).
| | Non-switching N=11 | Switching N=19 |
| --- | --- | --- |
| Median PFS, mo (95%CI) | 3.6 (1.5-7.4) | 2.1 (1.2-4.8) |
| HR (95%CI) 　　　　 　　 1.33 (0.61-2.89) 　　 　　　　　 p = 0.47 | | |
| | Non-switching N=29 | Switching N=5 |
| --- | --- | --- |
| Median PFS, mo (95%CI) | 4.2 (2.9-7.0) | 2.2 (1.4- Not Reached) |
| HR (95%CI) 　　　　 　　 1.36 (0.47-3.96) 　　 　　　　　 　　 p = 0.57 | | |
Number at risk
Number at risk
11
29
6
5
4
2
2
1
0
4
Non-switching
11
1
0
0
0
Non-switching
5
1
1
1
1
1
0
0
0
19
3
1
0
0
0
Switching
Switching
