# Supplementary material for: Disease progression status during initial immune checkpoint inhibitor (ICI) affects the clinical outcome of ICI retreatment in advanced non‐small cell lung cancer patients
Source: Cancer Med. 2023 Apr 16;12(11):12388–401. doi: 10.1002/cam4.5939 (PMC10278515; doi:10.1002/cam4.5939)
Supplement: Supplementary file 2 — Table S1. [file CAM4-12-12388-s001.docx]

**Supplementary Table 1. Initial ICI treatment**

| **Factors, n (%)** |  | **Overall (n = 64)** | | **PD group (n = 30)** | | **Without PD group**  **(n = 34)** | |
| --- | --- | --- | --- | --- | --- | --- | --- |
| **Initial ICI regimen** | Pembrolizumab | 21 | (32.8) | 5 | (16.7) | 16 | (47.1) |
|  | Nivolumab | 17 | (26.6) | 9 | (30.0) | 8 | (23.5) |
|  | Atezolizumab | 7 | (10.9) | 5 | (16.7) | 2 | (5.9) |
|  | Nivolumab+Ipilimumab | 3 | (4.7) | 0 | (0.0) | 3 | (8.8) |
|  | Durvalumab | 8 | (12.5) | 5 | (16.7) | 3 | (8.8) |
|  | Pembro+Chemo | 3 | (4.7) | 3 | (10.0) | 0 | (0.0) |
|  | Nivo+Chemo | 3 | (4.7) | 2 | (6.7) | 1 | (2.9) |
|  | Atezo+Chemo | 2 | (3.1) | 1 | (3.3) | 1 | (2.9) |

Abbreviations: Atezo, atezolizumab; Chemo, chemotherapy; ICI, immune checkpoint inhibitor; Nivo, nivolumab; Ipi, ipilimumab; Pembro, pembrolizumab.

**Supplementary Table 2. Immune-related adverse events during initial and ICI retreatment in patients with a previous history of ICI discontinue due to irAEs.**

|  | **irAEs during initial ICI n = 28** | | | | | | | | | |  | **irAEs during ICI rechallenge n = 28** | | | | | | | | | | | |
| --- | --- | --- | --- | --- | --- | --- | --- | --- | --- | --- | --- | --- | --- | --- | --- | --- | --- | --- | --- | --- | --- | --- | --- |
| **irAEs** | **Total** | | **Gr1/2** | | **Gr3/4** | | **corticosteroids** | | **leading to discontinue** | |  | **Total** | | **Gr1/2** | | **Gr3/4** | | **corticosteroids** | | **leading to discontinue** | | **Relapse** | |
|  |  |  |  |  |  |  |  |  |  |  |  |  |  |  |  |  |  |  |  |  |  |  |  |
| **Any irAEs, n(%)** | 28 | (100.0) | 16 | (57.1) | 12 | (42.9) | 28 | (100.0) | 28 | (100.0) |  | 10 | (35.7) | 7 | (25.0) | 3 | (10.7) | 8 | (28.6) | 7 | (25.0) | 8 | (28.6) |
| **Pneumonitis, n(%)** | 12 | (42.9) | 10 | (35.7) | 2 | (7.1) | 12 | (42.9) | 12 | (42.9) |  | 6 | (21.4) | 5 | (17.9) | 1 | (3.6) | 5 | (17.9) | 5 | (17.9) | 4 | (14.3) |
| **Colitis, n(%)** | 8 | (28.6) | 5 | (17.9) | 3 | (10.7) | 7 | (25.0) | 5 | (17.9) |  | 3 | (10.7) | 1 | (3.6) | 2 | (7.1) | 3 | (10.7) | 2 | (7.1) | 3 | (10.7) |
| **Hypo/hyper thyroidism, n(%)** | 5 | (17.9) | 5 | (17.9) | 0 | 0.0 | 0 | 0.0 | 2 | (7.1) |  | 1 | (3.6) | 1 | (3.6) | 0 | (0.0) | 0 | (0.0) | 0 | (0.0) | 0 | (0.0) |
| **Rash, n(%)** | 5 | (17.9) | 5 | (17.9) | 0 | 0.0 | 1 | (3.6) | 1 | (3.6) |  | 0 | (0.0) | 0 | (0.0) | 0 | (0.0) | 0 | (0.0) | 0 | (0.0) | 0 | (0.0) |
| **Hepatitis, n(%)** | 3 | (10.7) | 1 | (3.6) | 2 | (7.1) | 2 | (7.1) | 2 | (7.1) |  | 0 | (0.0) | 0 | (0.0) | 0 | (0.0) | 0 | (0.0) | 0 | (0.0) | 0 | (0.0) |
| **Adrenal insufficiency, n(%)** | 3 | (10.7) | 2 | (7.1) | 1 | (3.6) | 3 | (10.7) | 1 | (3.6) |  | 0 | (0.0) | 0 | (0.0) | 0 | (0.0) | 0 | (0.0) | 0 | (0.0) | 0 | (0.0) |
| **Arthritis, n(%)** | 2 | (7.1) | 1 | (3.6) | 1 | (3.6) | 2 | (7.1) | 2 | (7.1) |  | 0 | (0.0) | 0 | (0.0) | 0 | (0.0) | 0 | (0.0) | 0 | (0.0) | 0 | (0.0) |
| **Encephalitis, n(%)** | 1 | (3.6) | 0 | (0.0) | 1 | (3.6) | 1 | (3.6) | 1 | (3.6) |  | 0 | (0.0) | 0 | (0.0) | 0 | (0.0) | 0 | (0.0) | 0 | (0.0) | 0 | (0.0) |
| **Pericarditis, n(%)** | 1 | (3.6) | 0 | (0.0) | 1 | (3.6) | 1 | (3.6) | 1 | (3.6) |  | 0 | (0.0) | 0 | (0.0) | 0 | (0.0) | 0 | (0.0) | 0 | (0.0) | 0 | (0.0) |
| **Infusion reaction, n(%)** | 1 | (3.6) | 1 | (3.6) | 0 | (0.0) | 0 | (0.0) | 0 | (0.0) |  | 1 | (3.6) | 1 | (3.6) | 0 | (0.0) | 0 | (0.0) | 0 | (0.0) | 1 | (3.6) |
| **AMY elevation, n(%)** | 1 | (3.6) | 1 | (3.6) | 0 | (0.0) | 0 | (0.0) | 1 | (3.6) |  | 0 | (0.0) | 0 | (0.0) | 0 | (0.0) | 0 | (0.0) | 0 | (0.0) | 0 | (0.0) |
| **Diabetes mellitus, n(%)** | 1 | (3.6) | 0 | (0.0) | 1 | (3.6) | 0 | (0.0) | 0 | (0.0) |  | 0 | (0.0) | 0 | (0.0) | 0 | (0.0) | 0 | (0.0) | 0 | (0.0) | 0 | (0.0) |
